# Antidepressant, Anxiolytic and Neuroprotective Activities of Two Zinc Compounds in Diabetic Rats

**DOI:** 10.3389/fnins.2019.01411

**Published:** 2020-01-21

**Authors:** Christiane Leite Cavalcanti, Maria Conceição Rodrigues Gonçalves, Adriano Francisco Alves, Emmanuel Veríssimo de Araújo, Jader Luciano P. Carvalho, Priscilla Paulo Lins, Raquel Coutinho Alves, Naís Lira Soares, Liana Clebia Morais Pordeus, Jailane Souza Aquino

**Affiliations:** ^1^Programa de Pós Graduação em Ciências da Nutrição, Universidade Federal da Paraíba, João Pessoa, Brazil; ^2^Laboratório de Nutrição Experimental, Universidade Federal da Paraíba, João Pessoa, Brazil; ^3^Laboratório de Patologia, Universidade Federal da Paraíba, João Pessoa, Brazil

**Keywords:** anxiety, depression, supplementation, zinc, diabetic rats

## Abstract

Behavioral disorders affect most diabetic patients and Zinc (Zn) has been used among adjuvant therapies for involvement in the etiology of depression and anxiety, however, the results are still controversial. The objective of this study was to compare the antidepressant, anxiolytic and neuroprotective activity of the supplementation of two Zn compounds in an animal model of Diabetes Mellitus type 1 (DM1). Thirty-eight (38) adult rats were randomized into four groups: Control (C; *n* = 8); Diabetic (D; *n* = 10); Diabetic Zn Sulfate Supplement (DSZ; *n* = 10) and Diabetic Zn Gluconate Supplement (DGZ; *n* = 10). The DSZ group received Zn sulfate supplementation and the DGZ group received Zn gluconate supplementation at a dose of 15 mg/kg for 4 weeks. Data (mean ±SEM) were analyzed by the Mann–Whitney test with a significance level of *p* < 0.05. The results indicate that Zn gluconate supplementation in diabetic animals presented an antidepressant effect demonstrated through the results obtained in the Forced Swim Test, and neuroprotective effect by attenuating alterations in the cerebral cortex; while Zn sulfate supplementation in diabetic animals showed an anxiolytic effect demonstrated by the results obtained in the open field test and the elevated plus maze test. Considering the set of results, supplementation with both zinc compounds showed neurobehavioral benefits in diabetic animals with different effects depending on the type of anion associated with Zn.

## Introduction

Diabetes mellitus type 1 (T1DM) is a chronic disease characterized by insulin deficiency due to pancreatic β-cell loss and leads to hyperglycemia (American Diabetes Association, 2017). Diabetes Mellitus (DM) is a public health problem due to the large number of people affected and the consequences of the disease, that contribute to decreasing the quality of life of patients (American Diabetes Association, 2017). Studies have shown that DM is related to an increased prevalence of psychiatric disorders, among which depression and anxiety (Rotella and Mannucci, 2013; Petrak et al., 2015) are noteworthy.

Depression is a common, chronic, and disabling psychiatric disorder, strongly related to an anonymous state (Strandberg et al., 2014) resulting in social and economic burden, as well as enormous personal suffering and increased risk of mortality (Lépine and Briley, 2011). This disorder has been postulated to play a causal role in DM, and an increased risk has been reported in 60% of depressive individuals in developing this disease (Balhara, 2011). Similarly, the prevalence of anxiety disorders among diabetic patients is considerably higher compared to the general population (Huang et al., 2012).

In this context, it is imperative to seek adjuvants in treating diabetes and in preventing these disorders. Studies have related diabetes to zinc deficiency (Fernando and Zhou, 2015; Ranasinghe et al., 2015), even though this essential trace element is often present in the diets of diabetics in dietary sources such as eggs, cheese, meat, vegetables, whole grains, nuts and cereals, in addition to others such as several chemical form factors, the presence of absorption inhibitors or promoters, age and nutritional status of the individual may compromise their bioavailability in the body (Gibson, 2012). Zinc (Zn) plays substantial roles in inflammation suppression, oxidative stress reduction and in the correct functioning of lipid and glucose metabolism (Olechnowicz et al., 2018), especially in the brain, with higher concentrations being found in the hippocampus and amygdala regions (Grønli J. et al., 2013). Moreover, 300 enzymes are dependent on this trace element, many of them expressed in the central nervous system (Hagmeyer et al., 2015).

Zn deprivation affects brain homeostasis, leading to behavioral, cognitive and mental changes (Partyka et al., 2011; Szewczyk et al., 2011). In this sense, experimental and clinical observations have suggested that Zn is involved in the pathophysiology of depression and anxiety (Szewczyk et al., 2008; Mlyniec et al., 2014).

In fact, some studies suggest Zn as a protective agent against brain damage (Szewczyk, 2013), although it’s excess may produce cytotoxic effects (Sensi and Jeng, 2004; Plum et al., 2010). However, in clinical studies, the association between the prevalence of psychiatric disorders and zinc deficiency is controversial (Nguyen et al., 2009; Irmisch et al., 2010; Grønli O. et al., 2013).

Some studies with animals have shown that Zn deficiency increases symptoms which are similar to those of depression (Tassabehji et al., 2008; Mlyniec et al., 2012), while others have shown that Zn deficiency-like symptoms similar to depression appear to be reversed by antidepressant treatment (Whittle et al., 2008; Mlyniec and Nowak, 2012; Mlyniec et al., 2012). Other non-clinical studies have demonstrated a more expressive type of depressant behavior in animals with induced diabetes (Wayhs et al., 2013; De Morais et al., 2014).

Different Zn compounds such as oxides, hydroaspartate, chloride were intraperitoneally administered at varying doses (Joshi et al., 2012; Torabi et al., 2013; Satała et al., 2016) to test antidepressant properties in several behavioral tests, suggesting that Zn may potentiate the action of antidepressants, in addition to reducing their side effects so that their supplementation has a therapeutic effect (Siwek et al., 2010; Ranjbar et al., 2013). Zn sulfate and gluconate are the most common compounds administered orally (Capdor et al., 2013; Roohani et al., 2013), with the choice of this route being important in T1DM since patients already use injectable insulin. However, their actions so far have been poorly elucidated in behavioral tests in diabetic animals.

In this context, experimental studies involving Zn supplementation in diabetic rats substantially contribute to better understanding behavioral disorders, specifically depression and anxiety, as well as being essential for the safe prescription of Zn supplementation as an adjuvant treatment alternative. Thus, the present study aimed to compare the antidepressant, anxiolytic and neuroprotective activity of supplementing two Zn compounds in an animal model of Diabetes Mellitus Type 1 (T1DM).

## Materials and Methods

### Experimental Protocol

The experiments and protocols performed with the animals are in accordance with the principles recommended by Institute of Laboratory Animal Resources (2011). The protocols were approved by the Ethics Committee on Animal Use (CEUA) of the Federal University of Paraiba (UFPB), under no. 045/2015.

A total of 38 male adult Wistar (*Rattus norvegicus*, variety *albinus*) rats weighing 250 ± 30 g from Thomas George (UFPB) were used. The animals were housed in individual metabolic cages and maintained under standard lighting conditions (light/dark cycle, 12/12 h) and temperature (22 ± 2°C). Commercial ration (Presence, Paulínea, São Paulo) and filtered water were offered *ad libitum*. After 1 week of acclimatization, the animals were randomized into four groups: Control (C; *n* = 8); Diabetic (D; *n* = 10); Diabetic Zn sulfate supplemented (DSZ; *n* = 10) and Diabetic Zn gluconate supplemented (DGZ; *n* = 10) (Supplementary Figure S1). Data from healthy groups supplemented with zinc sulfate (SZ, *n* = 8) and zinc gluconate (GZ, *n* = 8) were presented as Supplementary Figures S2, S3.

### Induction of Diabetes Mellitus Type 1 (T1DM)

The procedure for chemical induction of diabetes in the animals of the D, DSZ and DGZ groups was performed after a 12-h fast. A solution of streptozotocin (STZ, Sigma-Aldrich, St. Louis, MO, United States) dissolved in citrate buffer (0.1 M, pH = 4.5) was intraperitoneally administered in a single dose of 50 mg/kg body weight (Santos et al., 2014). Group C intraperitoneally received equivalent doses of citrate buffer (0.1 M, pH = 4.5), without the addition of STZ. Three days after induction, animals that received glycemia greater than 250 mg/dL were considered diabetic and included in the experimental groups (Santos et al., 2014).

### Zinc Supplementation

The animals in the DSZ group received Zn sulfate supplementation and the animals in the DGZ group received Zn gluconate supplementation, both compounds orogastrically administered at a dose of 15 mg/kg body weight/day once daily for 4 weeks (Sapota et al., 2014). The dose choice used complied with the recommended maximum limit for rodents (National Academy of Sciences, 2001) and humans (Institute of Medicine, 2002) and it was calculated for each compound as a whole. The animals of groups C and D underwent the stress of a gavage with filtered water during the same period of the other groups.

### Behavioral Parameters

The animals of all groups were submitted to behavioral tests at the end of the 4 weeks of supplementation, and each test was performed on subsequent days. Ideal conditions were maintained for these tests: attenuation of noise levels, low illumination intensity and controlled temperature (±25°C). The animals were taken to the test room for acclimatization and adaptation half an hour before the experiments were performed.

#### Evaluation of Anxiolytic Activity

Anxiolytic activity was evaluated by the Elevated Plus Maze (EPM), in which behavioral conflict is based on the animal’s need to explore the environment and the potential danger it poses (Pellow et al., 1985).

The apparatus (Insight brand, Madeira, EP 151, Ribeirão Preto, São Paulo, Brazil) consists of a platform with two open arms (50 cm× 10 cm) perpendicular to two closed arms (50 cm× 10 cm× 50 cm), forming a cross and raised 50 cm from the ground. There is an acrylic border of 1 cm in height surrounding the open arms in order to avoid falls by the animals. Each animal was positioned in the center of the apparatus facing one of the closed arms and allowed to operate the apparatus for 5 min. Animal preference for open or closed arms was evaluated. An observer made the annotations regarding the percentage of entries in the open arms and closed arms, length of stay in each arms and total number of arm entries (open arm entries+close arm entries). The apparatus was cleaned with 10% ethyl alcohol and paper towels before and after the exposure of each animal, allowing it to dry naturally.

#### Motor Activity Assessment

The exploratory activity was evaluated by the Open Field (OF) test (Candland and Campbell, 1962), used for evaluating stimulant compounds or CNS depressants. The apparatus (Insight brand, EP 154, Ribeirão Preto, São Paulo, Brazil) consists of a circular, transparent acrylic box measuring 50 cm in height and 60 cm in diameter. The white acrylic floor (100 × 80 cm) is divided into concentric circles (15, 34, and 55 cm radius) and black radial lines forming 12 quadrants of similar areas. The animals were individually placed in the center of the device and the following behaviors were then observed during 5 min: a manual counter was used for recording ambulation (number of entries by the animal with four legs into any of the squares), rearing (number of times the animal is raised on its hind legs, perpendicular to the ground), grooming (number of times the animal self-cleans), immobility time (complete immobility of the animal) and number of fecal cakes. There was a specific observer for each evaluated behavior, totaling four properly trained observers. The apparatus was cleaned with 10% ethyl alcohol and paper towels before and after the exposure of each animal, allowing it to dry naturally.

#### Evaluation of Antidepressant Activity

Antidepressant activity was evaluated by the Forced Swim Test (FST) through observation of animal behavior in the face of an unconditioned stress situation (Porsolt et al., 1979).

A cylinder (30 cm in diameter and 50 cm in height) was used with water at a depth of 30 cm and temperature (25 ± 2°C). The animals were taken to the antechamber, where they remained for 30 min. After that time, the animals were individually placed in the cylinder with water and submitted to the test for 5 min in a single session, with their active (swimming and climbing) and passive behavior (immobility) evaluated (Slattery and Cryan, 2012). The evaluations were performed by two observers and filmed, then later transferred to a computer and analyzed in detail. At the end of the test the animals were dried with cloth towels. The water was exchanged before and after exposure of each animal, and the apparatus was cleaned with 10% ethyl alcohol, allowing it to dry naturally.

### Euthanasia

After 8 h fasting, the animals were intraperitoneally anesthetized with ketamine (25 mg/kg) associated with xylazine (25 mg/kg). The absence of reflexes was confirmed after anesthesia and the brains were collected for histological analysis.

### Histological Analysis of the Brain

The brains (right hemisphere) of each animal were sanitized with saline, fixed in 10% buffered formalin and stored in coded containers. The organs were processed according to the routine histopathological technique. Ten semi-serial cuts of 5 μm thick were obtained from the paraffin embedded material, following a cross-sectional plane to the analyzed organ of each animal. The obtained slides were stained using Hematoxylin and Eosin (H and E) technique, and the assembly was performed between lamina and laminula with synthetic resin (Entellan-Merck) for analysis in increasing lenses and photographed at 100x total magnification under an optical microscope (Motic BA 200, Kowloon, Hong Kong). The structural architectures of the organs and the presence, characteristic and intensity of possible inflammatory infiltrates were evaluated in these analyzes.

### Statistical Analysis

Data sets were tested for normality and homogeneity using tests of variance. Behavioral data (mean ±SEM) were analyzed by the Mann–Whitney test. The accepted level of significance was *p* < 0.05. The results were expressed as mean and standard error of the mean and were analyzed in Prism 6.0 software (GraphPad, San Diego, CA, United States).

## Results

### Behavioral Parameters of Diabetic Rats Supplemented With Two Different Zinc Compounds

**FIGURE 1:**
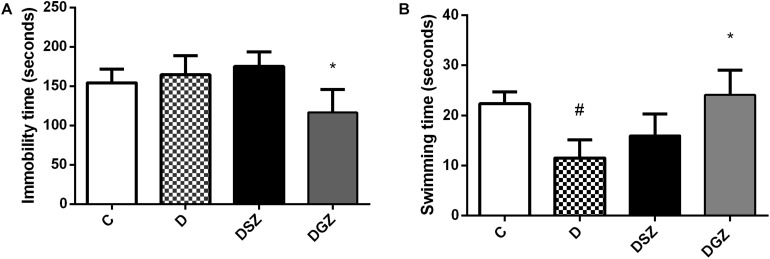
Evaluation of antidepressant activity using the Forced Swim Test (FST) in diabetic rats treated with two different zinc compounds (15 mg/kg). **(A)** Immobility time and **(B)** Swimming time. C, Control Group (*n* = 8); D, Diabetic Group (*n* = 10); DSZ (*n* = 10): Diabetic Group Supplemented with Zn Sulfate and DGZ (*n* = 10): Diabetic Group Supplemented with Zn Gluconate (*n* = 10). Mann–Whitney test (*p* < 0.05). ^∗^Significant difference compared with D group; ^#^significant difference compared with C group.

The study highlights the importance of zinc supplementation in the attenuated of common behavioral disorders, depression and anxiety in diabetes. Zn gluconate supplementation in diabetic animals reduced the time of passive behavior characterized by the animal’s immobility (116.6 ± 10.29 versus 164.4 ± 8.63 s, *p* < 0.05) and increased swimming time (24.12 ± 4.91 versus 11.5 ± 3.62 s, *p* < 0.05), both compared to group D (Figure 1). The active swimming behavior was similar between groups D and DSZ (*p* > 0.05).

**FIGURE 2:**
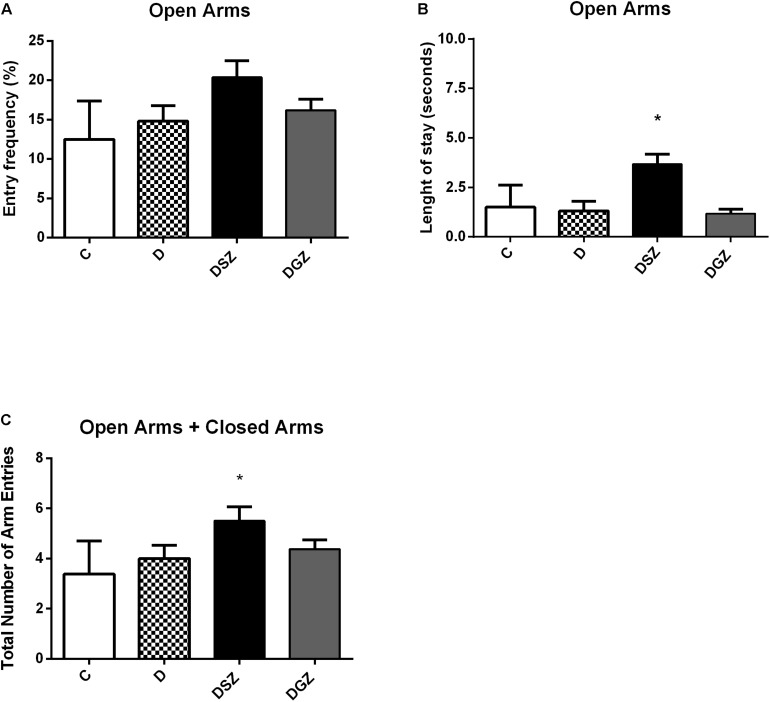
Evaluation of the anxiolytic activity using the Elevated Plus Maze (EPM) considering the percentage of length of stay, the percentage of number of entries in the open arms and the total number of arm entries (open arm entries + close arm entries) in diabetic rats treated with two different zinc compounds (15 mg/kg). **(A)** Entry frequency in open arms, **(B)** Length of stay in open arms and **(C)** Total number of arm entries. C, Control Group (*n* = 8); D, Diabetic Group (*n* = 10); DSZ (*n* = 10): Diabetic Group Supplemented with Zn Sulfate and DGZ (*n* = 10): Diabetic Group Supplemented with Zn Gluconate (*n* = 10). Mann–Whitney test (*p* < 0.05). ^∗^Significant difference compared with D group.

Regarding the anxious type behavior measured in the Elevated Labyrinth Plus test (Figure 2), the diabetic group supplemented with Zn sulfate (DSZ) had longer length of stay in the open arms (3.12 ± 0.39 versus 1.12 ± 0.39, *p* < 0.05), higher frequency of open arm entries (22.13 ± 2.99 versus 7.87 ± 2.96 s, *p* < 0.05) and greater total number of open plus closed arm entries compared to group D (5.50 ± 0.56 versus 4.00 ± 0.53, *p* < 0.05). Whereas the DGZ group presented shorter length of stay in the open arms and higher frequency of open arms entry than the group D (*p* < 0.05).

**FIGURE 3:**
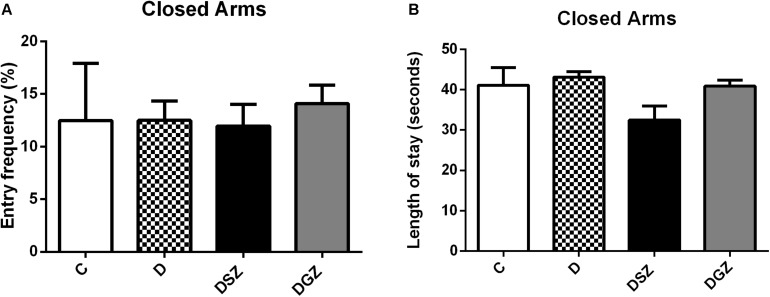
Evaluation of motor activity using the Open Field Test in diabetic rats treated with two different zinc compounds (15 mg/kg). **(A)** Entry frequency and **(B)** Length of stay in closed arms. C, Control Group (*n* = 8); D, Diabetic Group (*n* = 10); DSZ (*n* = 10): Diabetic Group Supplemented with Zn Sulfate and DGZ (*n* = 10): Diabetic Group Supplemented with Zn Gluconate (*n* = 10). Mann–Whitney test (*p* < 0.05).

The motor activity was measured using the Open Field (Figure 3). The DSZ group presented better responses as evidenced by greater ambulation (29.25 ± 0.90 versus 18.50 ± 2.24, *p* < 0.05) and shorter immobility time (3.12 ± 0.35 versus 6.87 ± 0.63 s, *p* < 0.05) compared to group D. The DGZ group had a shorter immobility time (3.87 ± 0.42 versus 6.87 ± 0.63 s, *p* < 0.05) compared to group D. In relation to the other evaluated parameters (rearing, grooming and number of fecal cakes) there were no differences (*p* > 0.05), so that these data were not included in the graphs (Supplementary Table S1).

### Histological Analysis of the Brain of Diabetic Rats Supplemented With Two Different Zinc Compounds

**FIGURE 4:**
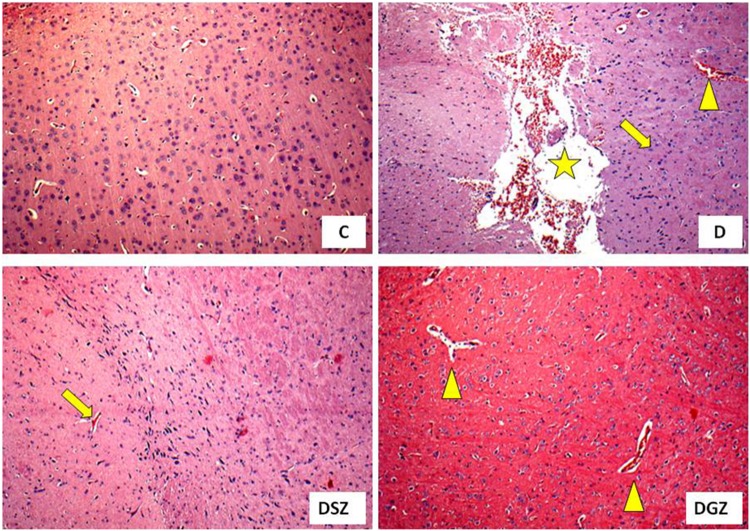
Histology of the cerebral cortex of diabetic rats treated with two different zinc compounds (H and E, × 100) C, control group (*n* = 8); D, diabetic group (*n* = 10), the arrow indicates ischemic neurons, the star indicates hemorrhage and the triangle indicates dilated vessels; DSZ, diabetic group supplemented with Zn Sulpfate (*n* = 10), the arrow indicates ischemic neurons; DGZ: Diabetic Group Supplemented with Zn Gluconate (*n* = 10), the triangle indicates dilated vessels.

Histology of the cerebral cortex of diabetic rats treated with two different zinc compounds is show in Figure 4, in order to associate these results with those of behavioral tests. It was observed that the group D animals presented cerebral degeneration characterized by the presence of ischemic neurons, hemorrhage and dilated vessels, whereas the DSZ group presented the presence of ischemic neurons, and animals from the DGZ group only had dilated vessels.

## Discussion

Diabetic animals had longer immobility time on the FST compared to diabetic and Zn Gluconate supplemented animals, thus reflecting their called “despair behavior” (Wayhs et al., 2010). The existence of DM is associated with anxiety disorders and increases the probability depression occurring (Golden et al., 2008; Huang et al., 2012). Our results suggest potential Zn Gluconate antidepressant activity evidenced by the shorter immobility time (*p* < 0.05) compared to the D and DSZ groups, considering that immobility time is used as an index of depressive behavior (Slattery and Cryan, 2012).

The swimming time parameter was used for evaluating locomotor activity and the results also showed a significant increase in the DGZ (*p* < 0.05) compared to the D group. The other active behavior parameter (climbing) did not present a statistically significant difference between groups (*p* > 0.05). In this sense, it is important to note that healthy animals supplemented with Zn did not obtain significant changes in behavior during the behavioral tests in our study, and therefore we can suggest that the beneficial effect of Zn supplementation only occurred in diabetic animals (Supplementary Figure S2).

A previous study (Szewczyk et al., 2011) found that immobility time on the FST was significantly higher in Zn-deficient mice, suggesting that a deficiency of this trace element causes an increase in behavior similar to depression. Hyperactivity of the glutamatergic system occurs in situations of Zn deficiency, thus generating depressive behavior, as demonstrated by Slattery and Cryan (2012). Animals using an antidepressant allied to an adequate or supplemented with Zn diet for 3 weeks showed reduced behavioral despair as measured by the FST (Tassabehji et al., 2008).

In recent decades, the Elevated Plus Maze (EPM) has established itself as the most classic animal model of exploratory behavior used in the laboratory. The animal explores both the open and closed arms of the labyrinth, but typically it will more frequently enter and will remain longer in the closed arms. However, a higher intensity of anxiety behavior equates to a lower preference for open arms and thus a higher predilection for closed arms (Ahn et al., 2013).

Our results demonstrated an increase in the length of stay and in the number of open arms (*p* < 0.05) in diabetic animals supplemented with Zn sulfate compared to the animals in groups D and DGZ, suggesting that this compound had an anxiolytic effect as confirmed by the longer stay in the open arms, and that the ambulation ability in these animals was preserved, indicating the ability of Zn to reduce anxiety without causing sedation.

In using the EPM to predict the potential anxiolytic activity of Zn hydroaspartate in rats and mice (Partyka et al., 2011) found an increase in the percentage of open arms entries in both species without significantly altering the length of stay in these arms. Zn chloride at different doses of Zn (15 and 20 mg/kg) promoted anxiolytic effects in supplemented rats, evidenced by the increase in the residence time and the greater number of open arms entries in the EPM (Joshi et al., 2012). Zn oxide supplementation at different doses (5 mg/kg and 10 mg/kg) resulted in an anxiolytic effect in the EPM test in rats (Torabi et al., 2013). However, a study on rats supplemented with Zn oxide at a higher dose (25 mg/kg) did not find a significant difference in the anxiety indexes assessed by the EPM (Amara et al., 2013).

In our study we evaluated five parameters: ambulation, latency time, rearing, grooming and fecal cakes. We realized that the abilities to explore the surrounding environment and spontaneous ability were severely impaired in group D, representing the behavioral and emotional changes that could mimic the clinical symptoms of depression. The increase in anxiety in OF is related to a decrease in locomotion and an increase in the peripheral area; an increased permanence in the central portion of the field indicates anxiolytic effect, whereas a decrease in the permanence in the central part of the device can be interpreted as an anxiogenic effect (Ahn et al., 2013).

The OF arena is considered a stressful environment for fear. More anxious and emotional animals tend to wander less and stay away from the central part of the arena in such conditions (Takeda et al., 2007; Strandberg et al., 2014). Regarding the evaluated parameters, we noticed that Zn sulfate supplementation in the DSZ group animals improved their locomotor activity in the OF test, increased the number of crossed quadrants and decreased their immobility time (*p* < 0.05), indicating a lower anxiety level compared to the D and DGZ groups. These results allow for suggesting that the locomotor activity was preserved after administrating Zn sulfate, so this Zn compound did not present sedative effect.

Were observed an increase in anxious behavior in Zn-deficient mice through less ambulation and less immobility during the OF test (Takeda et al., 2012). Rats fed a Zn-deficient diet for 2 weeks showed decreased ambulation and grooming, suggesting that anxiety-like behavior is increased in Zn deficiency (Xie et al., 2012). Behavioral changes may especially represent an effect of Zn deficiency and may be informative about the various pathways in which this trace element is an important functional factor in brain function (Hagmeyer et al., 2015).

In DM animal models, including the streptozotocin (STZ)-induced diabetic rodent model, abnormalities in the regulation of several neurotransmitters have been reported (Suzuki et al., 2012). Persisting hyperglycemia leads to impaired neurogenesis, decreased synaptic plasticity, undesired neuro-anatomical alterations, neurochemical deficits, and reduced neurotransmitter activity (Prabhakar et al., 2015). Zinc sulfate was not effective in improving the parameters evaluated in the FST and was effective in EPM and OF. While zinc gluconate was active in improving the parameters evaluated in FST and was not effective in EPM and OF. These data may indicate that the anion associated with Zn determines its antidepressant and anxiolytic activity. The antidepressant effect shown by Zn may be related to attenuation of the glutamatergic system by inhibition of N-methyl-D-aspartate (NMDA) receptor activity, as demonstrated in previous studies (Szewczyk et al., 2008, 2011; Mlyniec et al., 2012). Zn antidepressant and anxiolytic activities can also occur via modulating the serotonergic system through a complex mechanism not yet fully elucidated involving the participation of pre- and postsynaptic 5-HT_1__A_Rs (Satała et al., 2016). However, the mechanisms of how zinc-associated anions work in DM are not yet well understood.

Stress and anxiety have some parallels, as they appear to be genetically linked by sharing neurocircuits and common brain areas, including the prefrontal cortex, hippocampus, and amygdale (Sartori et al., 2011). In this sense, it is interesting to analyze some brain structures in search of connections between the role of Zn and the brain. In our study, we investigated the cerebral cortex of the animals and verified that the diabetic animals presented alterations characterized by the presence of ischemic neurons, hemorrhaging and dilated vessels, characterizing the oxidative stress of DM1. It is known that DM is associated with learning deficits and oxidative imbalances, neurophysiological and structural changes in cerebral cortex (Ates et al., 2014; Kim et al., 2015).

In our study, supplementation with the two Zn compounds showed beneficial effects on the cerebral cortex of diabetic animals, since these organs had more preserved structures with a reduction in ischemic neurons and hemorrhaging, suggesting a potential neuroprotective effect; especially of the Zn Gluconate compound at doses within the recommended maximum limit for rodents (National Academy of Sciences, 2001). Were found beneficial effects in the brains of healthy rats supplemented with Zn chloride (5 mg/kg) demonstrated by decreasing the degeneration and preservation of neuronal cells (Brocardo et al., 2007).

We have a limitation related to the absence of the plasma Zn dosage for verifying the deficiency in the animals. However, in view of the obtained only in diabetic animals and those supplemented with the two Zn compounds, it can be suggested that the resulting benefits may be due to the Zn deficiency in these animals, which may confirm a possible relation between DM1, Zn deficiency and behavioral changes.

## Conclusion

Considering the set of results, supplementation with both zinc compounds showed neurobehavioral benefits in diabetic animals with different effects depending on the type of anion associated with Zn: Zn gluconate had antidepressant and neuroprotective effect, while Zn sulfate had an anxiolytic effect, and further studies are needed to clarify how these mechanisms of action occur.

## Data Availability Statement

All datasets generated for this study are included in the article/Supplementary Material.

## Ethics Statement

The animal study was reviewed and approved by the Ethics Committee on Animal Use (CEUA) of the Federal University of Paraiba (UFPB), under no. 045/2015.

## Author Contributions

CC, MG, LP, and JA designed the study and the experiments. CC, AA, EA, JC, RA, PL, and NS carried out the experiments. CC, LP, and JA analyzed the data. CC, AA, and JA implemented the reagents, materials, and analysis tools. CC and JA wrote the manuscript.

## Conflict of Interest

The authors declare that the research was conducted in the absence of any commercial or financial relationships that could be construed as a potential conflict of interest.

## Supplementary Material

FIGURE S1 Experimental assay design.

FIGURE S2 Evaluation of antidepressant activity using the Forced Swim Test (FST) in healthy and diabetic rats treated with two different zinc compounds (15 mg/kg). C, control group (*n* = 8); SZ, healthy group supplemented with Zn Sulfate (*n* = 8); GZ, healthy group supplemented with Zn Gluconate (*n* = 8); D, diabetic group (*n* = 10); DSZ, diabetic group supplemented with Zn Sulfate and DGZ (*n* = 10): Diabetic Group Supplemented with Zn Gluconate (*n* = 10). Mann–Whitney test (*p* < 0.05). ^∗^Significant difference compared with D group; ^#^significant difference compared with C group.

FIGURE S3 Evaluation of motor activity using the Open Field (OF) Test in healthy and diabetic rats treated with two different zinc compounds (15 mg/kg). C, control group (*n* = 8); SZ, healthy group supplemented with Zn Sulfate (*n* = 8); GZ, healthy group supplemented with Zn Gluconate (*n* = 8); D, diabetic group (*n* = 10); DSZ, diabetic group supplemented with Zn Sulfate and DGZ (*n* = 10): Diabetic Group Supplemented with Zn Gluconate (*n* = 10). Mann-Whitney test (*p* < 0.05). ^∗^Significant difference compared with D group; ^#^significant difference compared with C group.

TABLE S1 Rearing, grooming and number of fecal cakes quantified in Open Field (OP) Test in healthy and diabetic rats treated with two different zinc compounds (15 mg/kg).
