# Supplementary figures and images for: Antidepressant, Anxiolytic and Neuroprotective Activities of Two Zinc Compounds in Diabetic Rats
Source: Front Neurosci. 2020 Jan 21;13:1411. doi: 10.3389/fnins.2019.01411 (PMC6985554; doi:10.3389/fnins.2019.01411)

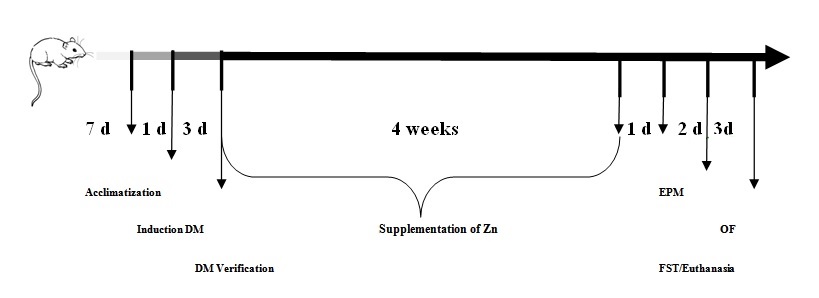

Supplement: FIGURE S1 — Experimental assay design. [file Image_1.jpg]
