# Supplementary material for: Antidepressant, Anxiolytic and Neuroprotective Activities of Two Zinc Compounds in Diabetic Rats
Source: Front Neurosci. 2020 Jan 21;13:1411. doi: 10.3389/fnins.2019.01411 (PMC6985554; doi:10.3389/fnins.2019.01411)
Supplement: TABLE S1 — Rearing, grooming and number of fecal cakes quantified in Open Field (OP) Test in healthy and diabetic rats treated with two different zinc compounds (15 mg/kg). [file Table_1.DOCX]

**Supplementary Table S1.** Rearing, grooming and number of fecal cakes quantified in Open Field (OP) Teste in healthy and diabetic rats treated with two different zinc compounds (15 mg/kg).

| **Parameters** | **Groups** | | | |
| --- | --- | --- | --- | --- |
|  | **C** | **D** | **DSZ** | **DGZ** |
| Rearing (seconds) | 8,16±2,06 | 8,75±1,86 | 8,34±1,30 | 8,00±1,21 |
| Grooming (seconds) | 13,28±1,61 | 13,35±1,50 | 12,3±1,18 | 12,8±1,21 |
| amount of fecal boli (units) | 2,8±1,30 | 3,0±1,24 | 3,2±1,45 | 2,6±1,74 |

C: Control Group (n=8); SZ: Healthy Group Supplemented with Zn Sulfate (n=8); GZ: Healthy Group Supplemented with Zn Gluconate (n=8); D: Diabetic Group (n= 10); DSZ: Diabetic Group Supplemented with Zn Sulfate and DGZ (n=10): Diabetic Group Supplemented with Zn Gluconate (n= 10). Mann-Whitney test (p <0.05).
